# Reduced dose direct oral anticoagulants compared with warfarin with high time in therapeutic range in nonvalvular atrial fibrillation

**DOI:** 10.1007/s11239-022-02763-w

**Published:** 2023-01-06

**Authors:** M. Jansson, S. Själander, V. Sjögren, F. Björck, H. Renlund, B. Norrving, A. Själander

**Affiliations:** 1 Department of Public Health and Clinical Medicine, Umeå University, Sundsvall, Sweden; 2 Uppsala Clinical Research Center, Uppsala University, Uppsala, Sweden; 3 Department of Clinical Sciences Lund, Neurology, Skåne University Hospital, Lund University, Lund, Sweden; 4 Cardiology Department, Sundsvall Hospital, 856 43 Sundsvall, Sweden

**Keywords:** Dabigatran, Rivaroxaban, Treatment outcome, Apixaban, Warfarin, Anticoagulants, Atrial fibrillation

## Abstract

Direct oral anticoagulants (DOACs) used in nonvalvular atrial fibrillation (NVAF) are dose-reduced in elderly and patients with impaired renal function. Only reduced dose dabigatran is concluded as having similar stroke risk reduction and lower risk of major bleeding than warfarin in the pivotal studies. In clinical practice, reduced dose is prescribed more often than expected making this an important issue. The objective of this study was to compare effectiveness and safety between reduced dose DOACs and high TTR warfarin treatment (TTR ≥ 70%) in NVAF. A Swedish anticoagulation registry was used in identifying eligible patients from July 2011 to December 2017. The study cohort consisted of 40,564 patients with newly initiated DOAC (apixaban, dabigatran, or rivaroxaban) (11,083 patients) or warfarin treatment (29,481 patients) after exclusion of 374,135 patients due to not being warfarin or DOAC naïve, not being prescribed reduced dose, having previous mechanical heart valve (MHV), or being under 18 years old. The median durations of follow up were 365, 419, 432 and 473 days for apixaban, dabigatran, rivaroxaban and warfarin, respectively. Warfarin TTR identified from Auricula was 70.0%. Endpoints (stroke and major bleeding) and baseline characteristics were collected from hospital administrative registers using ICD-10 codes. Cohorts were compared using weighted adjusted Cox regression after full optimal matching based on propensity scores. DOACs are associated with lower risk of major bleeding (HR with 95% CI) 0.85 (0.78–0.93), intracranial bleeding HR 0.64 (0.51–0.80), hemorrhagic stroke HR 0.68 (0.50–0.92), gastrointestinal bleeding HR 0.81 (0.69–0.96) and all-cause stroke HR 0.87 (0.76–0.99), than warfarin. Apixaban and dabigatran are associated with lower risk of major bleeding, HR 0.70 (0.63–0.78) and HR 0.80 (0.69–0.94), and rivaroxaban is associated with lower risk of ischemic stroke, HR 0.73 (0.59–0.96), with higher major bleeding risk, HR 1.31 (1.15–1.48), compared to warfarin. Apixaban is associated with higher all-cause mortality compared to warfarin, HR 1.12 (1.03–1.21). DOACs are associated with lower risk of major bleeding and all-cause stroke, than high quality warfarin treatment, with exception of rivaroxaban that carried higher risk of major bleeding and lower risk of stroke or systemic embolism. In this large observational registry-based NVAF cohort, DOACs are preferred treatment in patients with indication for DOAC dose reduction, even in a high TTR setting.

**Supplementary Information:**

The online version contains supplementary material available at 10.1007/s11239-022-02763-w.

## Introduction

Atrial fibrillation (AF) is one of the most common cardiac arrhythmias and the prevalence is increasing steadily, partly due to enhanced screening [1, 2]. A retrospective registry study with data from Swedish hospital administrative registries determined the Swedish prevalence of AF to be 2.9% in adults, undiscovered atrial fibrillation as well as atrial fibrillation treated in primary care only excluded [3].

AF is a strong and independent risk factor for stroke and increases the risk of stroke by near fivefold [2, 4]. Additionally, AF is associated with higher mortality in patients afflicted with ischemic stroke [5].

In patients with AF, Vitamin K antagonists (VKAs), e.g. warfarin, have been the standard in anticoagulative treatment for the last 50 years [6]. OAC with warfarin is superior to aspirin and placebo and warfarin therapy reduces the risk of stroke by two-thirds in patients with non-valvular atrial fibrillation (NVAF) [7–9]. Although anticoagulation with warfarin increases the risk of hemorrhagic stroke, the reduction of ischemic stroke risk using warfarin therapy outweighs the risk of bleeding, even for elderly and frail patients with frequent falls [10–13].

There are several limitations associated with treatment with VKAs, including genetic variations in metabolism, inertia in action onset and offset and various interactions with drugs and food [6, 14, 15]. Furthermore, additional factors such as dementia, major depression, active cancer, poverty, chronic liver disease, longer distance to care and frequent hospital admissions influence quality of treatment [16, 17]. The need for monitoring and dose-adjusting the VKA therapy as wells as concerns regarding patient compliance are common reasons for withholding therapy. Underuse of OACs for stroke prevention is common [18, 19]. Direct oral anticoagulants (DOACs) are the newest addition to the OAC arsenal. DOACs including apixaban, edoxaban, dabigatran and rivaroxaban, are all superior or non-inferior to warfarin in reducing the risk of stroke while at the same time having a similar or lower risk of bleeding, as concluded by both pivotal studies and later observational studies [20–25].

Dose reduction of DOAC therapy is indicated in patients with reduced renal function or frail elderly, as defined by creatinine levels, age and weight in the pivotal studies [20–23]. However, only dabigatran was tested in a separate study arm that compared reduced dose with warfarin. Treatment with reduced dose dabigatran was concluded as having similar stroke risk reduction as warfarin while having lower risk of major bleeding [20].

DOACs are prescribed in reduced dose to a higher proportion of patients than expected from clinical trials. There are a handful of previous studies concerning reduced dose DOACs, mainly focusing on evaluation of the appropriateness of the prescribed dose, showing a consistent tendency to underdosing [26–33]. An American study consisting of 5,738 patients concluded that amongst DOAC‐treated patients, 9.4% were underdosed, and 3.4% were overdosed [33].

Our objective was to compare the effectiveness and safety between reduced dose DOACs and high TTR warfarin treatment (≥ 70%) in NVAF.

## Material and methods

The study was approved by the Ethics Review Board in Umeå (2015/142-31).

The study population was identified from Auricula, the Swedish anticoagulation quality registry. Auricula contains information on OAC therapy on about 135,000 patients that are treated with OAC. The Auricula registry was started in 2006 and includes both inpatient and outpatient diagnoses of AF. The registry data includes OAC treatment indication, start and stop dates for therapy, prescribed OAC dosage, INR values of patients on warfarin, and creatinine levels in patients treated with DOACs. At the time of data extraction, Auricula was used in the majority of the Swedish healthcare regions, and the registry contained information on approximately half of the Swedish patients with DOAC treatment. Warfarin mean TTR was 70.5% during 2017 [34].

**Fig. 1:**
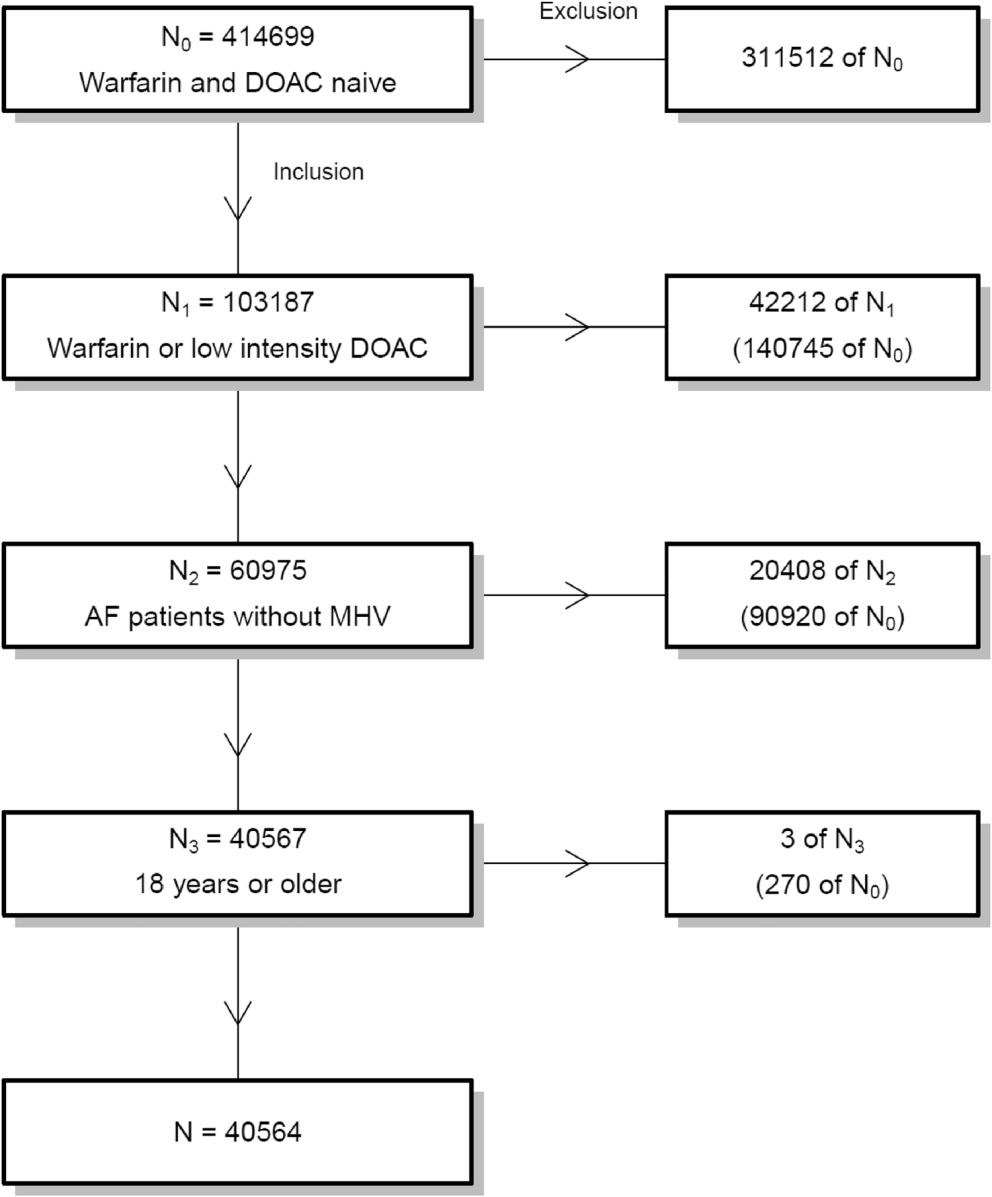
Flow chart of cohort formation. Patients in the registry were 414,699. Then, 311,512 patients were excluded due to not being warfarin or DOAC naïve. Following, 42,212 patients were excluded due to not being treated with warfarin or reduced dose DOAC, 20,408 patients were excluded due to indication mechanical heart valve (MHV) and lastly 3 patients younger than 18 years were excluded, forming a study cohort of 40,564 patients

The study included patients with NVAF who were registered in the Auricula registry and prescribed a new treatment period with apixaban, dabigatran, rivaroxaban or warfarin between July 1, 2011, and December 31, 2017. Only patients treated with reduced dose DOAC (apixaban 2.5 mg BD, dabigatran 110 mg BD or rivaroxaban 15 mg OD) were included. This study does not include patients treated with edoxaban since the prescription frequency of that specific medication was low and the average duration of treatment was short at the moment of registry data retrieval. Patients with ongoing OAC therapy at January 1, 2011, were excluded. Nonetheless, a previous OAC treatment period was allowed. New treatment was defined as initiating OAC treatment for a patient for the first time. Patient previously treated with VKA was accepted. However, switching between DOACs was not allowed. The exposure definition in this study is “as-treated”, considering the entire treatment period. Patients with valvular AF were excluded (Fig. 1).

Outcome events (end points) in this study included all-cause stroke, systemic embolism, ischemic stroke, hemorrhagic stroke, all-cause mortality, myocardial infarction and major bleeding. Major bleeding was defined as bleeding that were fatal or required hospital care. However, erythrocyte transfusion was not included in the major bleeding end point, due to missing information on the number of transfused units of red blood cells when using ICD-10-codes. Ischemic stroke and intracerebral hemorrhage (ICH) were identified in Riksstroke while death was identified from the Cause of Death registry. In this study, hemorrhagic stroke was defined as either ICH or subarachnoid hemorrhage (SAH). ICH as endpoint was included in hemorrhagic stroke, intracranial bleeding, all-cause stroke and major bleeding, but also an endpoint by itself. Succinctly, outcome events in this study included up to three types of bleeding per patient; intracranial (including SAH, ICH, subdural and epidural bleeding), gastrointestinal and/or bleeding of other location. However, the analysis only included the first event of each type of end point. Briefly, all outcomes during follow up that required specialist care or was fatal, as well as stroke or intracranial bleeding of any severity, were included in the study and analyzed. ICD-10-codes used in outcome identification are listed in Table 1, Appendix A.

Most events during follow up and previous medical illnesses were collected from the Swedish National Patient Registry (NPR). The NPR is a health care administrative registry that was founded in 1964. The registry contains information from both specialized inpatient and outpatient care and is superintended by the Swedish National Board of Health and Welfare. The degree of coverage is almost 100% regarding inpatient care. Of discharged patients, merely 0.8% are missing an ICD-10 (International Classification of Diseases 10th edition) code as main diagnosis. External validation determined a positive predictive value (PPV) of a diagnosis in the NPR to be as high as 85–95% [35]. The diagnosis AF has a PPV of 97% [36]. Past medical event history and co-morbidities were identified using ICD-10 codes listed in Table 2, Appendix A.

The Swedish Stroke Register (Riksstroke), which is a Swedish national quality registry of stroke care, was used for identification of events of ischemic stroke and ICH during the study period considering its higher validity for stroke related diagnoses when comparing to the NPR. Additionally, in NPR there is a possibility for the same stroke incident to be registered multiple times, a problem that does not exist in Riksstroke. Riksstroke has an estimated coverage of 89% in 2017, with a stable coverage during the time period of the study [37].

The Swedish Cause of Death registry was used in collection of date of death of deceased persons.

### Statistical methods

The statistical analysis of end points was made with weighted adjusted Cox regression using standard errors from sandwich estimators. Determination of weights were made using full optimal matching based on propensity scores. To specify, an optimal matching of two groups creates clusters of individuals from both groups in a way that minimize some metric globally. No individuals are discarded in full optimal matching. In the analysis, full optimal matchings between apixaban, rivaroxaban, dabigatran and warfarin were created using the R package optmatch. The Mahalanobis distance between a set of covariates were used as base for the matchings (Table 2) [38]. The clusters were weighted such the total weight equaled the study population size and that each cluster was balanced. Succinctly, the weighted groups were more comparable in baseline covariates, and the parametric analysis were less vulnerable to any model misspecification [39]. Lastly, we performed a sensitivity analysis, excluding patients with inappropriate dosing, not following the Summaries of Product characteristics (SPC) recommendations.

## Results

### Cohort description

The study included in total 40,564 patients with NVAF. Of these, 11,083 were treated with DOAC in reduced dose (6,592 patients on apixaban, 2,162 on dabigatran and 2,329 on rivaroxaban). Remaining 29,481 patients were treated with warfarin (Table 1). The mean age in the warfarin group was 75 years, in the dabigatran group 81.4 years, in the apixaban group 85.5 years and in the rivaroxaban group 83.5 years. In the warfarin group, 57.8% were male, with corresponding figures of 47.7%, 39.6% and 46.6% in the dabigatran, apixaban and rivaroxaban groups, respectively. The median CHA_2_DS_2_VASc score was 3 in all of the treatment groups.

**Table 1:** Baseline clinical characteristics of treatment groups prior to weighting, presented as n (%)

	Apixaban (n = 6,592)	Dabigatran (n = 2,162)	Rivaroxaban (n = 2,329)	Warfarin (n = 29,481)
Duration of follow-up				
Median, days (IQR)	365 (142–684)	419 (138–1,033)	432 (156–847)	473 (105–1,352)
Demographic characteristics				
Age (years), mean (IQR)	85.5 (80.8–89.6)	81.4 (76.2–85.4)	83.5 (78.3–88.2)	75 (67.8–81.9)
Male gender	2,608 (39.6)	1,032 (47.7)	1,086 (46.6)	17,054 (57.8)
Prior medical conditions				
Anemia	451 (6.8)	79 (3.7)	138 (5.9)	942 (3.2)
Cancer	910 (13.8)	289 (13.4)	367 (15.8)	3,223 (10.9)
Chronic obstructive pulmonary disease	504 (7.6)	145 (6.7)	148 (6.4)	1,677 (5.7)
Congestive heart failure	1,076 (16.3)	201 (9.3)	334 (14.3)	2,842 (9.6)
Dementia	90 (1.4)	30 (1.4)	25 (1.1)	121 (0.4)
Diabetes	575 (8.7)	151 (7.0)	257 (11.0)	2,527(8.6)
Excessive alcohol use	70 (1.1)	36 (1.7)	36 (1.5)	545 (1.8)
Fall	1,779 (27)	442 (20.4)	549 (23.6)	4,037 (13.7)
Hypertension	1,176 (17.8)	348 (16.1)	440 (18.9)	4,058 (13.8)
Renal failure	282 (4.3)	18 (0.83)	84 (3.6)	1,119 (3.8)
Ischemic stroke	1,150 (17.4)	433 (20)	351 (15.1)	3,864 (13.1)
Stroke or TIA	1,545 (23.4)	560 (25.9)	500 (21.5)	5,077 (17.2)
TIA	587 (8.9)	203 (9.4)	207 (8.9)	1,665 (5.6)
Vascular disease	1,608 (24.4)	380 (17.6)	531 (22.8)	5,541 (18.8)
Liver disease	63 (1.0)	19 (0.9)	18 (0.8)	246 (0.8)
Myocardial infarction	1,321 (20.0)	310 (14.3)	432 (18.5)	4,546 (15.4)
Prior bleeding				
Major bleeding	1,615 (24.5)	443 (20.5)	515 (22.1)	4,915 (16.7)
Intracranial bleeding	202 (3.1)	74 (3.4)	61 (2.6)	353 (1.2)
Intracerebral bleeding	152 (2.3)	58 (2.7)	42 (1.8)	258 (0.9)
Traumatic intracranial bleeding	72 (1.1)	22 (1.0)	24 (1.0)	127 (0.4)
Gastrointestinal bleeding	476 (7.2)	101 (4.7)	139 (6)	1,202 (4.1)
Other bleeding	1,117 (16.9)	319 (14.8)	356 (15.3)	3,688 (12.5)
Prior cardiovascular intervention				
PCI	146 (2.2)	41 (1.9)	42 (1.8)	481 (1.6)
PCI with stenting	12 (0.2)	4 (0.2)	15 (0.6)	72 (0.2)
Indication for treatment				
Primary stroke prevention in AF	5,969 (90.5)	1,871 (86.5)	2,164 (92.9)	26,949 (91.4)
Secondary prevention after stroke in AF	611 (9.3)	288 (13.3)	160 (6.9)	2,407 (8.2)
Secondary prevention PAE in AF	12 (0.2)	3 (0.1)	5 (0.2)	125 (0.4)
CHA_2_DS_2_-VASc score				
Median	3 (3–5)	3 (2–4)	3 (3–4)	3 (2–4)

*PCI* percutaneous coronary intervention

*PAE* peripheral arterial embolism

CHA_2_DS_2_-VASc score = 1 point each (except where noted) for history of congestive heart failure, hypertension, aged ≥ 75 years (2 points), diabetes mellitus, previous stroke or TIA or thromboembolism (2 points), vascular disease, female sex and aged 65–74 years

There were no significant differences in patient baseline characteristics between warfarin and reduced dose DOACs after full optimal matching (Table 2).

**Table 2:** Baseline clinical characteristics in weighted cohorts, presented as % except for age

	DOAC reduced dose (n = 11,083)	Warfarin (n = 29,481)	Comparison (Std)
Demographic characteristics			
Age (years), mean (IQR)	84.3 (79.5–88.7)	84.4 (79.3–88.7)	0.0017
Male gender	42.6	42.6	0
Prior medical conditions			
Congestive heart failure	14.5	17.0	− 0.0650
Diabetes	8.9	9.1	− 0.0092
Excessive alcohol use	1.3	1.2	− 0.0005
Hypertension	17.7	19.8	− 0.0550
Liver disease	0.9	0.76	0.0017
Myocardial infarction	18.6	20.8	− 0.0530
Vascular disease	22.7	25.4	− 0.0590
Renal failure	3.5	5.1	− 0.0870
Stroke/TIA	23.5	24.7	− 0.0280
Prior bleeding			
Gastrointestinal bleeding	6.5	6.9	− 0.0005
Intracranial bleeding	3.0	2.6	0.0220
Other bleeding	16.2	16.6	− 0.0150
Prior cardiac intervention			
PCI	2.1	2.6	− 0.0350

*PCI* percutaneous coronary intervention

### Outcomes

#### Warfarin vs. reduced dose DOACs

**Fig. 2:**
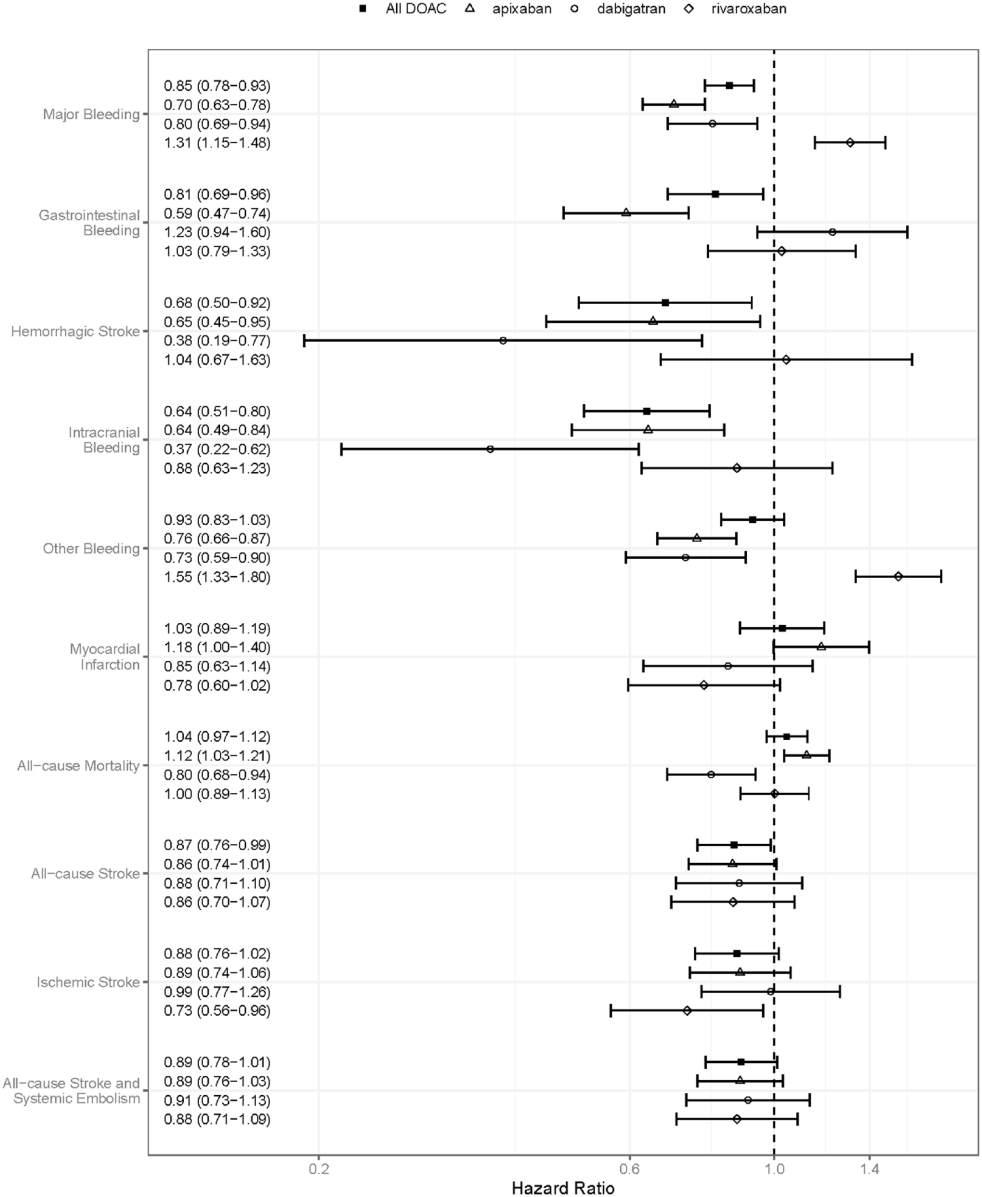
Hazard ratios with confidence intervals in comparisons after full match weighted Cox regression for all DOAC as a group as well as individually, compared with matched warfarin controls

After matching, there were significant differences in rate of major bleeding, gastrointestinal bleeding and intracranial bleeding between reduced dose DOACs as a group and warfarin treatment with HR 0.85 (95% CI 0.78–0.93), HR 0.81 (0.69–0.96) and HR 0.64 (0.51–0.80) respectively. Also, the rate of hemorrhagic stroke and all-cause stroke differed significantly, HR 0.68 (0.50–0.92) and HR 0.87 (0.76–0.99) respectively (Fig. 2 and Table 3.)

**Table 3:** Primary outcomes presented as events per treatment year in warfarin and reduced dose DOAC treatment groups

Outcome	Warfarin			DOAC reduced dose			Comparison
	Time (years)	Events	Rate	Time (years)	Events	Rate	rr
Major bleeding	12,079.4	1014.3	0.084	14,443.9	1023	0.071	0.84
Gastrointestinal bleeding	12,678.8	277.4	0.022	15,110.1	264	0.017	0.80
Hemorrhagic stroke	12,821.6	93.8	0.007	12,821.6	93.8	0.007	0.66
Intracranial bleeding	12,781.4	184.1	0.014	15,238.4	137	0.009	0.62
Other bleeding	12,249.3	604.1	0.049	14,642.2	661	0.014	0.92
Myocardial infarction	12,619.0	330.0	0.026	15,029.3	370	0.025	0.94
All-cause mortality	12,828.4	1382.4	0.110	15,281.5	1648	0.110	1.00
All-cause stroke	12,464.9	473.0	0.038	14,931.0	462	0.031	0.82
Ischemic stroke	12,476.0	355.1	0.028	14,955.3	350	0.023	0.82
All-cause stroke and systemic embolism	12,450.9	483.9	0.039	14,916.8	485	0.033	0.84

#### Warfarin vs. rivaroxaban in reduced dose

There were no differences between reduced dose rivaroxaban and warfarin when comparing events of all-cause mortality, myocardial infarction, all-cause stroke and all-cause stroke and systemic embolism. However, reduced dose rivaroxaban treatment was associated with a lower risk of ischemic stroke, HR 0.73 (0.56–0.96) (Fig. 2).

Considering bleeding, there were no differences when comparing events of intracranial bleeding, gastrointestinal bleeding or hemorrhagic stroke. The rates of major bleeding and other bleeding were higher on reduced dose rivaroxaban treatment compared with warfarin with HR 1.31 (1.15–1.48) and HR 1.55 (1.33–1.80) respectively (Fig. 2).

#### Warfarin vs. dabigatran in reduced dose

There were no differences between reduced dose dabigatran and warfarin when comparing events of myocardial infarction, ischemic stroke, all-cause stroke and all-cause stroke and systemic embolism. However, reduced dose dabigatran was associated with significantly lower risk of all-cause mortality (Fig. 2).

Reduced dose dabigatran was associated with significantly lower risk of major bleeding, intracranial bleeding, other bleeding and hemorrhagic stroke, HR 0.80 (0.68–0.94), HR 0.37 (0.22–0.62), HR 0.73 (0.59–0.90) and HR 0.38 (CI 0.19–0.77) respectively. The gastrointestinal bleeding rate did not differ compared with warfarin (Fig. 2).

#### Warfarin vs. apixaban in reduced dose

There were no differences between reduced dose apixaban and warfarin when comparing events of myocardial infarction, ischemic stroke, all-cause stroke, and all-cause stroke and systemic embolism (Fig. 2). Reduced dose apixaban was associated with significantly higher risk of all-cause mortality, HR 1.12 (1.03–1.21) (Fig. 2).

Furthermore, patients treated with reduced dose apixaban had a significantly lower rate of major bleeding, gastrointestinal bleeding, intracranial bleeding, other bleeding and hemorrhagic stroke, HR 0.70 (0.63–0.78), HR 0.59 (0.47–0.74), HR 0.64 (0.49–0.84), HR 0.76 (0.66–0.87) and HR 0.65 (0.45–0.95) respectively (Fig. 2).

### Sensitivity analysis

A sensitivity analysis was performed with a guideline subgroup, excluding 3,745 (33.8%) patients with inappropriate dosing, i.e. prescribed reduced dose DOAC despite indication for standard dosing according to the SPC. This analysis did not change the overall findings (Appendix A, Fig. 2).

## Discussion

Reduced dosing of DOACs is a frequent clinical concern as it impacts the effectiveness and safety of the anticoagulation therapy. This study was designed to examine differences in effectiveness and safety when comparing reduced dose DOACs with high-quality warfarin treatment, through matched analyses.

In our cohort study, routine practice registry-based data show that patients with AF have a lower risk of major bleeding when treated with reduced dose apixaban or dabigatran when comparing with warfarin, while rivaroxaban patients both had higher risk of major bleeding and lower risk of ischemic stroke compared with warfarin. The study did not show any statistically significant difference in effectiveness considering all-cause stroke and systemic embolism when comparing reduced dose dabigatran, apixaban or rivaroxaban to warfarin. However, as a group, reduced dose DOAC carried lower risk of all cause stroke.

When comparing type of bleeding, treatment with dabigatran was associated with lower risk of major bleeding, intracranial bleeding, hemorrhagic stroke and other bleeding than patients treated with warfarin. The hazard ratio is as low as 0.37 when comparing the risk of intracranial bleeding between the dabigatran and warfarin treatment group. Reduced dose apixaban treatment was associated with lower risk of major bleeding, gastrointestinal bleeding, intracranial bleeding, hemorrhagic stroke and other bleeding when comparing to warfarin. The hazard ratio is 0.64 when comparing apixaban intracranial bleeding risk to warfarin treatment. There was no difference in the risk of ischemic stroke when comparing dabigatran and apixaban with warfarin treatment. When comparing the reduced dose rivaroxaban treatment group with warfarin, the tables have turned. Rivaroxaban treatment was associated with higher risk of major bleeding (where the risk of other bleeding stands out as significantly higher), while also correlating with lower risk of ischemic stroke, when compared with high quality warfarin treatment. This could either be due to actual differences in the risk of bleeding between studied DOACs, or a leaning for the clinicians to choose rivaroxaban due to the once daily dosing regimen in patients with perceived worse compliance to prescribed medication and therefore also a higher risk of bleeding. Also, the relatively lower dose adjustment in rivaroxaban patients compared to apixaban patients (20 mg to 15 mg OD vs 5 mg to 2.5 mg BD) could mean a relatively higher dosing, and anticoagulant effect, in rivaroxaban patients compared to patients with other DOACs in reduced dosing regimen.

Dabigatran in reduced dose was associated with lower all-cause mortality than patients treated with warfarin while treatment with reduced dose apixaban was associated with a significantly higher risk of all-cause mortality than warfarin treatment. Similarly, the lower all-cause mortality seen in the dabigatran treatment group, and higher the like in the apixaban group, might be due to confounding by indication that remains despite the full optimal matching. Previous studies show increased all-cause mortality in patients with subtherapeutic dosing of apixaban [40]. However, in this study, the increased all-cause mortality of apixaban remains after sensitivity analysis for inappropriate dosing. There could be a reluctance to use dabigatran in elderly patients with impaired renal function while apixaban might be a more common choice in frail patients which could confound the results in a way that we have not been able to correct for. Also, previous studies show that patients receiving apixaban had a higher CHA2DS2-VASc score, higher cardiovascular risk, and more comorbid conditions (Charlson score) than those receiving other DOACs [41],

Previous findings in clinical trials meta-analyses conclude DOACs as being non-inferior or superior to warfarin in prevention of stroke and systemic embolism as well as having similar or lower risk of bleeding. However, there is no discrimination of standard or reduced dosing [20–22, 42–46]. Several observational studies comparing DOACs and warfarin have reproduced these findings [24, 25, 47–49]. Furthermore, meta-analyses and observational studies comparing DOACs vs. warfarin indicate a lower risk of major bleeding with use of dabigatran and apixaban compared with use of rivaroxaban [23, 49–52].

There are studies comparing effectiveness and safety between different reduced dose DOACs [53, 54]. However, studies comparing reduced dose DOAC and warfarin treatment are few [55–59]. One study consisting of aggregated data abstracted from Phase III trials shows that patients eligible for reduced dose DOACs had a favorable benefit-harm profile when compared to warfarin treatment, with lower risk of major bleeding, hemorrhagic stroke, intracranial hemorrhage and fatal bleeding. However, the study was conducted as a meta-analysis by subgroups in randomized controlled trials (RCTs) and therefore had heterogeneity in studied populations. Also, RCTs are not fully representative of a real-world population in terms of external validity, limiting the relevance of findings to clinical practice to some extent [55]. However, a large Japanese observational health claims data study consisting of 73,989 patients shows a significantly lower risk of major intracranial hemorrhage when comparing all DOACs with warfarin, and also significantly lower risk of ischemic stroke in the apixaban and rivaroxaban groups when compared with warfarin, albeit without information on warfarin treatment quality defined as TTR [56]. Also, a large American observational health claims data study including 76,940 patients showed a significantly lower risk of stroke/systemic embolism and major bleeding in favor of apixaban when compared with warfarin. However, information on warfarin treatment quality was missing and previous studies have shown a suboptimal TTR in UC clinical practices where patients having the highest risk of stroke or bleeding were least likely to be in therapeutic range [60, 61]. Lastly, a meta-analysis which compared DOACs with warfarin in atrial fibrillation presented a separate analysis on reduced dosing, showing similar stroke prophylaxis efficacy and a non-significant reduction in major bleeding [62]. The results of this study partly confirm and partly differ from previous observational registry-based studies, showing a favorable safety profile with a lower risk of intracranial bleeding and major bleeding. The study shows no significant difference in ischemic stroke risk when comparing apixaban and dabigatran treatment with warfarin, while rivaroxaban carries a higher risk of major bleeding and intracranial bleeding, but significantly lower risk of ischemic stroke. The quality of warfarin treatment was high in the current study. Time in therapeutic range (TTR) in the warfarin treatment group was 70%. Our study is, to our knowledge, the first to compare apixaban, dabigatran and rivaroxaban with high quality warfarin treatment in an observational registry-based study with full optimal matching design. With current guidelines recommending DOACs as first-line anticoagulant therapy for patients with NVAF, this study contributes additional findings from a clinical routine practice setting concerning the individual benefit-harm profiles associated with reduced dose DOACs compared with high quality warfarin treatment.

### Limitations

This study is non-randomized with a retrospective design, with an inherent risk of bias. Also, no conclusions regarding causality are possible due to the study design. However, this observational registry-based study expands the external validity of previous RCTs. Diagnosis codes may be incorrectly coded, and there is no data on diagnoses from the primary health part of outpatient care. However, a previous study has shown only small effects of primary health care diagnoses when determining CHA2DS2-VASc score [63]. Furthermore, we are not able to measure non-prescription drugs since there is no data regarding this in the national databases. However, there is no obvious reason that the use of alcohol or NSAID would differ between the different treatment groups as is required to bias the results. In addition, the matching of the treatment cohorts has limitations. A specific DOAC may have been chosen as treatment for a specific patient for reasons that cannot be adjusted for using ICD-10 codes. The study focuses on reduced dosing of DOACs but appropriate use of reduced DOAC dose has not been verified and dosing is based on decisions from individual physicians. Differences in the proportions of incorrect reduced dose between the DOACs could affect a comparison of their clinical safety and effectiveness. However, sensitivity analysis shows no difference in overall findings when excluding patients with inappropriate dosing. Also, the end point major bleeding is defined as bleeding that is fatal or requires hospitalization. There is no use of classifications such as BARC 3-5, TIMI or GUSTO due to lack of data in the available registers. Following this, some of the bleedings, e.g., gastrointestinal bleedings, may have been misdefined since they required hospitalization but still not fulfilled major bleeding criteria according to definitions above. Lastly, patients with previous exposure to anticoagulation may have lower bleeding risk when compared to the anticoagulation naïve. However, the patient proportion with prior treatment is small and therefore this limitation does not affect the comparisons.

## Conclusions

In this large observational registry-based NVAF cohort, comparison of treatment with reduced dose DOACs and high TTR warfarin treatment show a favorable effectiveness and safety profile of clinical significance for reduced dose DOACs which are associated with strongly reduced risk of intracranial bleeding. Treatment with DOACs in reduced dose are associated with lower risk of major bleeding and all-cause stroke when compared with a warfarin treated cohort. When comparing specific DOACs with warfarin, apixaban and dabigatran are not more effective in stroke and embolism prevention, although they are associated with lower risk of major bleeding, while treatment with rivaroxaban is associated with more effective prevention of ischemic stroke but with higher risk of major bleeding instead. Apixaban is associated with higher all-cause mortality. In patients with NVAF, reduced dose DOACs are associated with a clinically significant reduced risk of major bleeding as well as all-cause stroke and is the preferred treatment choice over warfarin, even in a high TTR setting.

## Supplementary Information

Below is the link to the electronic supplementary material.

Supplementary file1 (DOCX 508 kb)

## Abbreviations

DOACs: Direct oral anticoagulants
NVAF: Non-valvular atrial fibrillation
AF: Atrial fibrillation
VKAs: Vitamin K antagonists
OAC: Oral anticoagulant
NPR: National Patient Registry
PPV: Positive predictive value
ATC: Anatomic Therapeutic Chemical
SAH: subarachnoid hemorrhage
ICH: Intracerebral hemorrhage
PAE: Peripheral artery embolism

## References

[1] Schnabel RB, Yin X, Gona P, Larson MG, Beiser AS, McManus DD (2015). 50 year trends in atrial fibrillation prevalence, incidence, risk factors, and mortality in the Framingham Heart Study: a cohort study. Lancet.

[2] Go AS, Hylek EM, Phillips KA, Chang Y, Henault LE, Selby JV (2001). Prevalence of diagnosed atrial fibrillation in adults: national implications for rhythm management and stroke prevention: the AnTicoagulation and Risk Factors in Atrial Fibrillation (ATRIA) Study. JAMA.

[3] Friberg L, Bergfeldt L (2013). Atrial fibrillation prevalence revisited. J Intern Med.

[4] Wolf PA, Abbott RD, Kannel WB (1991). Atrial fibrillation as an independent risk factor for stroke: the Framingham Study. Stroke.

[5] Marini C, De Santis F, Sacco S, Russo T, Olivieri L, Totaro R (2005). Contribution of atrial fibrillation to incidence and outcome of ischemic stroke: results from a population-based study. Stroke.

[6] Plitt A, Ruff CT, Giugliano RP (2016). Non-vitamin K antagonist oral anticoagulants in atrial fibrillation. Hematol Oncol Clin N Am.

[7] Connolly SJ, Eikelboom J, Joyner C, Diener HC, Hart R, Golitsyn S (2011). Apixaban in patients with atrial fibrillation. N Engl J Med.

[8] Hart RG, Pearce LA, Aguilar MI (2007). Adjusted-dose warfarin versus aspirin for preventing stroke in patients with atrial fibrillation. Ann Intern Med.

[9] Hart RG, Pearce LA, Aguilar MI (2007). Meta-analysis: antithrombotic therapy to prevent stroke in patients who have nonvalvular atrial fibrillation. Ann Intern Med.

[10] Donze J, Clair C, Hug B, Rodondi N, Waeber G, Cornuz J (2012). Risk of falls and major bleeds in patients on oral anticoagulation therapy. Am J Med.

[11] Man-Son-Hing M, Nichol G, Lau A, Laupacis A (1999). Choosing antithrombotic therapy for elderly patients with atrial fibrillation who are at risk for falls. Arch Intern Med.

[12] Sjalander A, Engstrom G, Berntorp E, Svensson P (2003). Risk of haemorrhagic stroke in patients with oral anticoagulation compared with the general population. J Intern Med.

[13] Friberg L, Rosenqvist M, Lip GY (2012). Net clinical benefit of warfarin in patients with atrial fibrillation: a report from the Swedish atrial fibrillation cohort study. Circulation.

[14] Ruff CT, Giugliano RP, Braunwald E, Antman EM (2014). New oral anticoagulants in patients with atrial fibrillation—authors’ reply. Lancet.

[15] Ferder NS, Eby CS, Deych E, Harris JK, Ridker PM, Milligan PE (2010). Ability of VKORC1 and CYP2C9 to predict therapeutic warfarin dose during the initial weeks of therapy. J Thromb Haemost.

[16] Hylek EM, Skates SJ, Sheehan MA, Singer DE (1996). An analysis of the lowest effective intensity of prophylactic anticoagulation for patients with nonrheumatic atrial fibrillation. N Engl J Med.

[17] Rose AJ, Miller DR, Ozonoff A, Berlowitz DR, Ash AS, Zhao S (2013). Gaps in monitoring during oral anticoagulation: insights into care transitions, monitoring barriers, and medication nonadherence. Chest.

[18] Frankel DS, Parker SE, Rosenfeld LE, Gorelick PB (2015). HRS/NSA 2014 survey of atrial fibrillation and stroke: gaps in knowledge and perspective, opportunities for improvement. Heart Rhythm.

[19] Gorin L, Fauchier L, Nonin E, Charbonnier B, Babuty D, Lip GYH (2011). Prognosis and guideline-adherent antithrombotic treatment in patients with atrial fibrillation and atrial flutter: implications of undertreatment and overtreatment in real-life clinical practice; the Loire Valley Atrial Fibrillation Project. Chest.

[20] Connolly SJ, Ezekowitz MD, Yusuf S, Eikelboom J, Oldgren J, Parekh A (2009). Dabigatran versus warfarin in patients with atrial fibrillation. N Engl J Med.

[21] Patel MR, Mahaffey KW, Garg J, Pan G, Singer DE, Hacke W (2011). Rivaroxaban versus warfarin in nonvalvular atrial fibrillation. N Engl J Med.

[22] Granger CB, Alexander JH, McMurray JJ, Lopes RD, Hylek EM, Hanna M (2011). Apixaban versus warfarin in patients with atrial fibrillation. N Engl J Med.

[23] Giugliano RP, Ruff CT, Braunwald E, Murphy SA, Wiviott SD, Halperin JL (2013). Edoxaban versus warfarin in patients with atrial fibrillation. N Engl J Med.

[24] Sjogren V, Bystrom B, Renlund H, Svensson PJ, Oldgren J, Norrving B (2017). Non-vitamin K oral anticoagulants are non-inferior for stroke prevention but cause fewer major bleedings than well-managed warfarin: a retrospective register study. PLoS ONE.

[25] Larsen TB, Skjoth F, Nielsen PB, Kjaeldgaard JN, Lip GY (2016). Comparative effectiveness and safety of non-vitamin K antagonist oral anticoagulants and warfarin in patients with atrial fibrillation: propensity weighted nationwide cohort study. BMJ.

[26] Barra ME, Fanikos J, Connors JM, Sylvester KW, Piazza G, Goldhaber SZ (2016). Evaluation of dose-reduced direct oral anticoagulant therapy. Am J Med.

[27] Deguchi I, Takao M (2019). Reduced doses of direct oral anticoagulants in ischemic stroke patients with nonvalvular atrial fibrillation. J Stroke Cerebrovasc Dis.

[28] Howerton MA, Suhrie EM, Gennari AS, Jones N, Ruby CM (2019). Evaluation of direct oral anticoagulant dosing and monitoring in two geriatric outpatient clinics. Sr Care Pharm.

[29] Huang ZC, Li CQ, Liu XY, Cao ZC, Jia HY, Dong Y (2020). Efficacy and safety of direct oral anticoagulants in patients with atrial fibrillation and liver disease: a meta-analysis and systematic review. Cardiovasc Drugs Ther.

[30] Yao X, Shah ND, Sangaralingham LR, Gersh BJ, Noseworthy PA (2017). Non-vitamin K antagonist oral anticoagulant dosing in patients with atrial fibrillation and renal dysfunction. J Am Coll Cardiol.

[31] Steinberg BA, Shrader P, Pieper K, Thomas L, Allen LA, Ansell J (2018). Frequency and outcomes of reduced dose non-vitamin K antagonist anticoagulants: results from ORBIT-AF II (The Outcomes Registry for Better Informed Treatment of Atrial Fibrillation II). J Am Heart Assoc.

[32] de Almeida JPHC, Martinho AS, Girão A, Barreiro I, Milner J, Ferreira MJV (2020). Novel anticoagulants in an older and frail population with atrial fibrillation: the effect of inappropriate dosing on clinical outcomes. Eur Geriatr Med.

[33] Steinberg BA, Shrader P, Thomas L, Ansell J, Fonarow GC, Gersh BJ (2016). Off-label dosing of non-vitamin K antagonist oral anticoagulants and adverse outcomes: the ORBIT-AF II Registry. J Am Coll Cardiol.

[34] Juhlin T (2017) Auricula årsrapport 2017. Annual report of Auricula 2017 **(in Swedish)**. https://www.ucr.uu.se/auricula/kvalitetsregister/arsrapporter/arsrapporter/auricula-arsrapport-20172018

[35] Ludvigsson JF, Andersson E, Ekbom A, Feychting M, Kim JL, Reuterwall C (2011). External review and validation of the Swedish national inpatient register. BMC Public Health.

[36] Smith JG, Platonov PG, Hedblad B, Engstrom G, Melander O (2010). Atrial fibrillation in the Malmo Diet and Cancer study: a study of occurrence, risk factors and diagnostic validity. Eur J Epidemiol.

[37] Register TSS (2017) Riksstrokes årsrapport 2017. Annual report of Riksstroke 2017 **(in Swedish)**. http://www.riksstroke.org/sve/forskning-statistik-och-verksamhetsutveckling/forskning/arsrapporter/2018

[38] Hansen BB, OlsenKlopfer S (2006). Optimal full matching and related designs via network flows. J Comput Graph Stat.

[39] Ho DE, Imai K, King G, Stuart E (2007). Matching as nonparametric preprocessing for reducing model dependence in parametric causal inference. Polit Anal.

[40] Ashraf H, Agasthi P, Shanbhag A, Mehta RA, Rattanawong P, Allam M (2021). Long-term clinical outcomes of underdosed direct oral anticoagulants in patients with atrial fibrillation and atrial flutter. Am J Med.

[41] Ganse EV, Danchin N, Mahé I, Hanon O, Jacoud F, Nolin M (2020). Comparative safety and effectiveness of oral anticoagulants in nonvalvular atrial fibrillation: the NAXOS study. Stroke.

[42] Miller CS, Grandi SM, Shimony A, Filion KB, Eisenberg MJ (2012). Meta-analysis of efficacy and safety of new oral anticoagulants (dabigatran, rivaroxaban, apixaban) versus warfarin in patients with atrial fibrillation. Am J Cardiol.

[43] Capodanno D, Capranzano P, Giacchi G, Calvi V, Tamburino C (2013). Novel oral anticoagulants versus warfarin in non-valvular atrial fibrillation: a meta-analysis of 50,578 patients. Int J Cardiol.

[44] Dogliotti A, Paolasso E, Giugliano RP (2013). Novel oral anticoagulants in atrial fibrillation: a meta-analysis of large, randomized, controlled trials vs warfarin. Clin Cardiol.

[45] Lopez-Lopez JA, Sterne JAC, Thom HHZ, Higgins JPT, Hingorani AD, Okoli GN (2017). Oral anticoagulants for prevention of stroke in atrial fibrillation: systematic review, network meta-analysis, and cost effectiveness analysis. BMJ.

[46] Lamberts M, Staerk L, Olesen JB, Fosbol EL, Hansen ML, Harboe L (2017). Major bleeding complications and persistence with oral anticoagulation in non-valvular atrial fibrillation: contemporary findings in real-life Danish patients. J Am Heart Assoc..

[47] Yao X, Abraham NS, Sangaralingham LR, Bellolio MF, McBane RD, Shah ND (2016). Effectiveness and safety of dabigatran, rivaroxaban, and apixaban versus warfarin in nonvalvular atrial fibrillation. J Am Heart Assoc..

[48] Potpara TS, Lip GY (2017). Postapproval observational studies of non-vitamin K antagonist oral anticoagulants in atrial fibrillation. JAMA.

[49] Lip GY, Larsen TB, Skjoth F, Rasmussen LH (2012). Indirect comparisons of new oral anticoagulant drugs for efficacy and safety when used for stroke prevention in atrial fibrillation. J Am Coll Cardiol.

[50] Schneeweiss S, Gagne JJ, Patrick AR, Choudhry NK, Avorn J (2012). Comparative efficacy and safety of new oral anticoagulants in patients with atrial fibrillation. Circ Cardiovasc Qual Outcomes.

[51] Mantha S, Ansell J (2012). An indirect comparison of dabigatran, rivaroxaban and apixaban for atrial fibrillation. Thromb Haemost.

[52] Graham DJ, Reichman ME, Wernecke M, Hsueh YH, Izem R, Southworth MR (2016). Stroke, bleeding, and mortality risks in elderly medicare beneficiaries treated with dabigatran or rivaroxaban for nonvalvular atrial fibrillation. JAMA Intern Med.

[53] Staerk L, Gerds TA, Lip GYH, Ozenne B, Bonde AN, Lamberts M (2018). Standard and reduced doses of dabigatran, rivaroxaban and apixaban for stroke prevention in atrial fibrillation: a nationwide cohort study. J Intern Med.

[54] Jansson M, Själander S, Sjögren V, Renlund H, Norrving B, Själander A (2020). Direct comparisons of effectiveness and safety of treatment with apixaban, dabigatran and rivaroxaban in atrial fibrillation. Thromb Res.

[55] Wang KL, Lopes RD, Patel MR, Büller HR, Tan DS, Chiang CE (2019). Efficacy and safety of reduced-dose non-vitamin K antagonist oral anticoagulants in patients with atrial fibrillation: a meta-analysis of randomized controlled trials. Eur Heart J.

[56] Kohsaka S, Katada J, Saito K, Jenkins A, Li B, Mardekian J (2020). Safety and effectiveness of non-vitamin K oral anticoagulants versus warfarin in real-world patients with non-valvular atrial fibrillation: a retrospective analysis of contemporary Japanese administrative claims data. Open Heart.

[57] Huang HY, Lin SY, Cheng SH, Wang CC (2018). Effectiveness and safety of different rivaroxaban dosage regimens in patients with non-valvular atrial fibrillation: a nationwide, population-based cohort study. Sci Rep.

[58] Larsen TB, Gorst-Rasmussen A, Rasmussen LH, Skjøth F, Rosenzweig M, Lip GY (2014). Bleeding events among new starters and switchers to dabigatran compared with warfarin in atrial fibrillation. Am J Med.

[59] Cheng WH, Chao TF, Lin YJ, Chang SL, Lo LW, Hu YF (2019). Low-dose rivaroxaban and risks of adverse events in patients with atrial fibrillation. Stroke.

[60] Li XS, Deitelzweig S, Keshishian A, Hamilton M, Horblyuk R, Gupta K (2017). Effectiveness and safety of apixaban versus warfarin in non-valvular atrial fibrillation patients in “real-world” clinical practice. A propensity-matched analysis of 76,940 patients. Thromb Haemost.

[61] Pokorney SD, Simon DN, Thomas L, Fonarow GC, Kowey PR, Chang P (2015). Patients’ time in therapeutic range on warfarin among US patients with atrial fibrillation: results from ORBIT-AF registry. Am Heart J.

[62] Ruff CT, Giugliano RP, Braunwald E, Hoffman EB, Deenadayalu N, Ezekowitz MD (2014). Comparison of the efficacy and safety of new oral anticoagulants with warfarin in patients with atrial fibrillation: a meta-analysis of randomised trials. Lancet.

[63] Forslund T, Wettermark B, Wandell P, von Euler M, Hasselstrom J, Hjemdahl P (2013). Risk scoring and thromboprophylactic treatment of patients with atrial fibrillation with and without access to primary healthcare data: experience from the Stockholm health care system. Int J Cardiol.

