# Supplementary material for: Reduced dose direct oral anticoagulants compared with warfarin with high time in therapeutic range in nonvalvular atrial fibrillation
Source: J Thromb Thrombolysis. 2023 Jan 6;55(3):415–25. doi: 10.1007/s11239-022-02763-w (PMC10110706; doi:10.1007/s11239-022-02763-w)

# **Appendix A. Supplementary data**

**Table 1**. ICD-10-codes used in identifying outcomes

| Outcome | Criteria |
| --- | --- |
| All-cause stroke and systemic embolism | NPR^a^: I60, RS^b^: I61, I63, I64, NPR: I74 |
| All-cause stroke | NPR: I60, RS: I61, I63, I64 |
| Ischaemic stroke | RS: I63 |
| Haemorrhagic stroke | NPR: I60, RS: I61 |
| Major bleeding | Any of intracranial, gastrointestinal, or other bleeding |
| Intracranial bleeding | NPR: I60, RS: I61, NPR: I62, S064–066 |
| Gastrointestinal bleeding | I850, I983, K250, K252, K254, K256, K260, K262, K264, K266, K270, K272, K274, K276, K280, K282, K284, K286, K625, K920–922 |
| Other bleeding | H113, H313, H356, H431, H450, H922, I312, J942, M250, N02, N501A, N938, N939, N950, R04, R319, R58, T810, D500, D508, D509, D629 |
| Myocardial infarction | I21, I22 |

^a^National Patient Registry, ^b^Swedish Stroke Register

**Table 2**. ICD-10-codes used in medical history

| Item in medical history | Criteria |
| --- | --- |
| History of fall | ≥2 occurrences of W00–W19 |
| Cancer | C00–C26, C30–C41, C43–C58, C60–C97 |
| Stroke | I60, I61, I63, I64, I69 |
| Transient ischaemic attack (TIA) | G45 (except G454) |
| Stroke or TIA | I63, I64, I69, G45 (except G454) |
| Hypertension | I10–I13, I15 |
| Congestive heart failure | I110, I130, I132, I50 |
| Diabetes | E10–14 |
| Myocardial infarction | I21, I252 |
| Ischaemic heart disease | I20–I23, I241, I248, I249, I251, I252, I255, I256, I258, I259 |
| Chronic obstructive pulmonary disease | J43, J44 |
| Anaemia | D50, D510, D513, D518, D519, D52, D53, D55, D560–562, D568, D569, D570–D572, D588, D589, D59–D64 |
| Major bleeding | D629, I60–I62, I850, I983, K250, K252, K254, K256, K260, K262, K264, K266, K270, K272, K274, K276, K280, K282, K284, K286, K625, K920, K922 |
| Gastrointestinal bleeding | I850, I983, K250, K252, K254, K256, K260, K262, K264, K266, K270, K272, K274, K276, K280, K282, K284, K286, K625, K920, K922 |
| Intracranial bleeding | I60–I62, S064–S066 |
| Cerebral haemorrhage | I60, I61 |
| Previous traumatic intracranial bleeding | S064–S066 |
| Renal failure | I120, I131, I132, N182–N185, N189, DR016, DR024, KAS00, KAS10, KAS20 |
| Excessive alcohol use | E244, F10, G312, G621, G721, I426, K292, K70, T51, Y90, Y91, K860, O354, Z714 |
| Dementia | F00–F03 |
| Liver disease | K70–K77, JJB, JJC |
| Vascular disease | I21, I22, I252, I70–I73 |
| PCI | Z955 |

**Figure 1**. Standardized differences when comparing DOACS and warfarin pre matching.


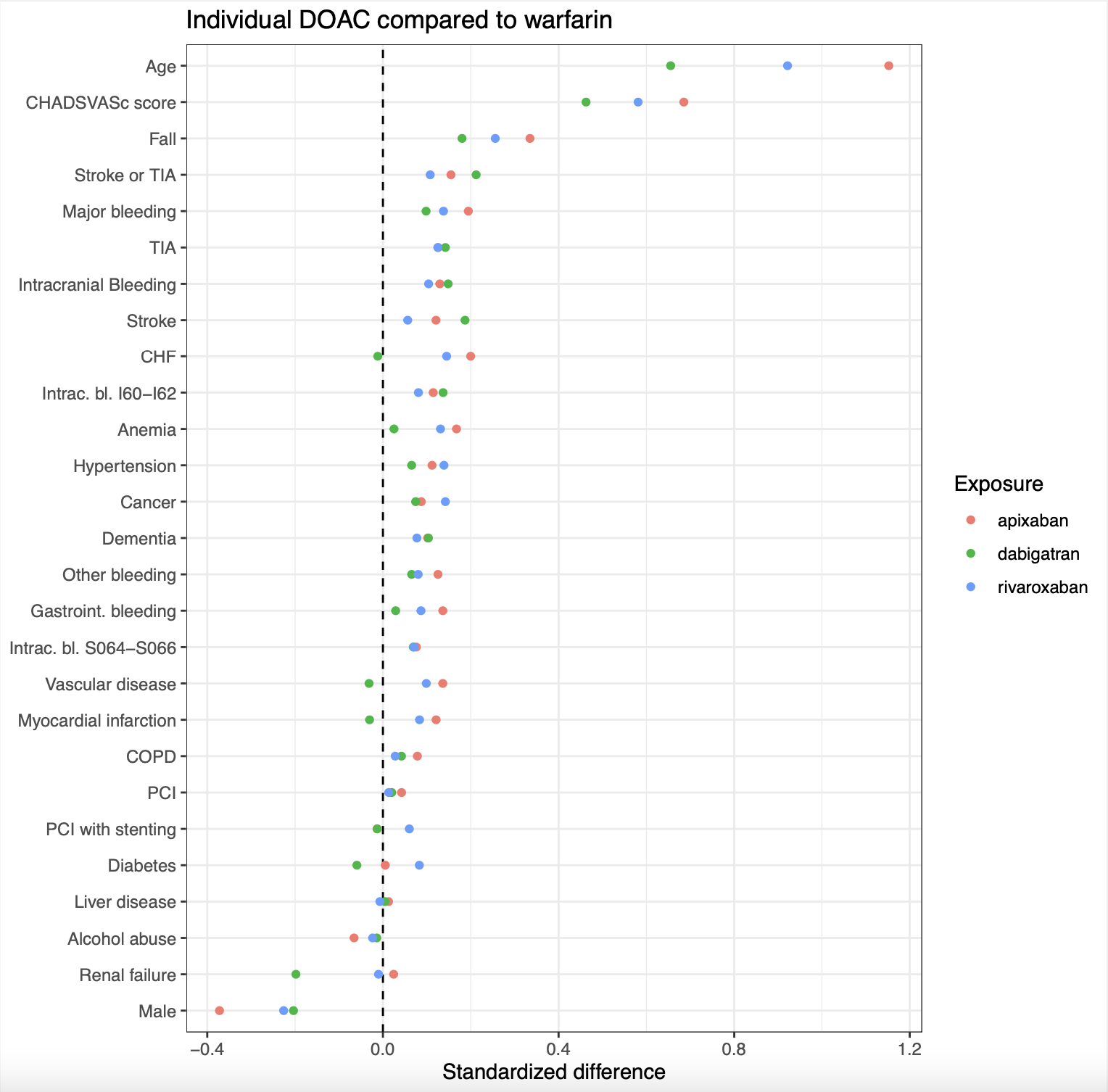


**Figure 2.** Sensitivity analysis – exclusion inappropriate dosed patients

Hazard ratios with confidence intervals in comparisons after full match weighted Cox regression for all DOAC as a group (n=11,082) and guideline subgroup (n=7,337), excluding 3,745 patients with inappropriate reduced dosing according to the SPC.


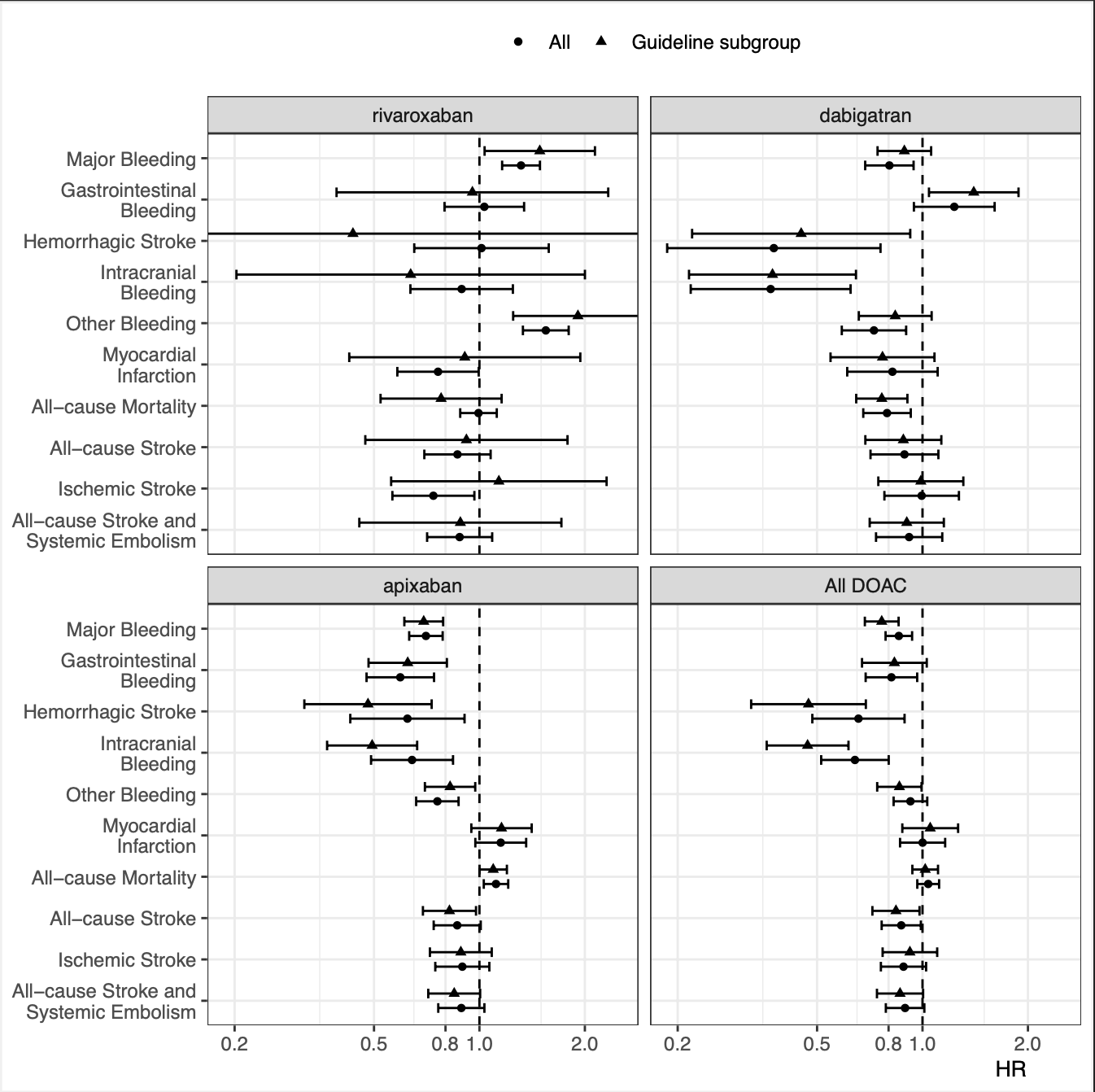

Supplement: Supplementary file 1 — Supplementary file1 (DOCX 508 kb) [file 11239_2022_2763_MOESM1_ESM.docx]
